# The Response of Mucosal Colonic Microbiota to Probiotic and Dietary Intervention In Vitro

**DOI:** 10.3390/microorganisms14020270

**Published:** 2026-01-23

**Authors:** Agnieszka Rudzka, Ondřej Patloka, Magdalena Płecha, Marek Zborowski, Renata Barczyńska-Felusiak, Tomasz Królikowski, Michał Oczkowski, Danuta Kołożyn-Krajewska, Dorota Zielińska

**Affiliations:** 1 Department of Dietetics and Food Studies, Faculty of Science and Technology, Jan Dlugosz University in Czestochowa, Al. Armii Krajowej 13/15, 42-200 Częstochowa, Poland; d.kolozyn-krajewska@ujd.edu.pl; 2 Department of Food Technology, Faculty of AgriSciences, Mendel University in Brno, Zemědělská 1, 61300 Brno, Czech Republic; xpatlok3@mendelu.cz; 3 Institute of Biochemistry and Biophysics, Polish Academy of Sciences, Pawińskiego 5a, 02-106 Warsaw, Poland; mk.plecha.ibb@gmail.com; 4 Institute of Evolutionary Biology, Faculty of Biology, Biological and Chemical Research Centre, University of Warsaw, Żwirki i Wigury 101, 02-089 Warsaw, Poland; 5 The Faculty of Medicine and Health Sciences, University of Applied Sciences in Nowy Sącz, Kościuszki 2G, 33-300 Nowy Sącz, Poland; mzborowski@ans-ns.edu.pl; 6 Department of Dietetics, Institute of Human Nutrition Sciences, Warsaw University of Life Sciences (WULS-SGGW), Nowoursynowska 159 C, 02-776 Warsaw, Poland; tomasz_krolikowski@sggw.edu.pl (T.K.); michal_oczkowski@sggw.edu.pl (M.O.); 7 Department of Gastronomic Technology and Food Hygiene, Institute of Human Nutrition Sciences, Warsaw University of Life Sciences (WULS-SGGW), Nowoursynowska 159 C, 02-776 Warsaw, Poland; dorota_zielinska@sggw.edu.pl

**Keywords:** Mucosal Simulator of Human Gastrointestinal Microbial Ecosystem, mucosal microbiota, dietary intervention, probiotic intervention, *Lacticaseibacillus rhamnosus* GG

## Abstract

Recently, the role of mucosal intestinal microbiota in human health has received increasing attention. Nevertheless, data on the response of this microbiota to various interventions remain limited. Here, we have employed the Mucosal Simulator of Human Gastrointestinal Microbial Ecosystem (M-SHIME^®^) and luminal SHIME^®^ (L-SHIME^®^) to examine mucosal microbiota responses to interventions that are known to impact the intestinal microbial community in humans and study relationships between the responses of mucosal and luminal microbiota. Specifically, we evaluated the effects of varying macronutrient levels over a 28-day standard, balanced dietary intervention and a parallel 14-day administration of *Lacticaseibacillus rhamnosus* GG. Observed shifts in mucosal microbiota in response to interventions differed significantly from those observed in luminal microbiota (*p* < 0.05). In particular, we found that the mucosal microbiota compared to luminal microbiota was more stable and that the abundance of several genera (i.e., *Subdoligranulum*, *Parabacteroides* and *Fusobacterium*) in the M-SHIME^®^ correlated positively with the intake of dietary macronutrients, especially protein, which was in line with results reported in previous human studies. This study demonstrates the reliability of advanced in vitro models in capturing diet-induced dynamics of the human mucosal microbiota, a compartment that remains understudied despite its critical role in intestinal immune regulation.

## 1. Introduction

Recent years have brought many studies focused on human mucosal microbiota. The mucosal layer is characterized by gradients in the permeability and oxygen concentration [1]. These conditions contribute to mucosa-associated microbiota being compositionally distinct from luminal microbiota [2]. Studies have shown that the mucosal samples tend to be enriched in Proteobacteria and Bacteroidetes and depleted in Firmicutes compared with the luminal specimens from the same donors [2,3]. Moreover, the ability of some bacterial species, such as *Akkermansia muciniphila* or *Bacteroides thetaiotaomicron*, to degrade mucins is thought to underlie their preferential association with the mucosal compartment of the intestinal ecosystem [4].

Despite the progress in mucosal microbiota research, the reports on its composition contain some conflicting information. Some authors noted that this community is uniform over the entire length of the intestines [5], while others showed large differences in samples taken from different compartments of the digestive tract [6]. These discrepancies may be attributed to differences in sample procurement and analytical protocols, but also to methodological challenges connected to invasive sampling required to access this part of the human microbial ecosystem.

Nevertheless, studies explaining the role of mucosal intestinal microbiota are very valuable. This part of the microbiota is in the closest proximity to the intestinal barrier, and hence its role in health maintenance and pathological conditions may be crucial [7]. In fact, it may be greater than the impact of so far exhaustively researched fecal microbiota [8,9]. For example, accumulating evidence indicates that this component of the human microbiota plays a pivotal role in maintaining intestinal barrier function, immune regulation, and the pathogenesis of inflammatory bowel disease [10].

The last two decades brought developments in the in vitro technology enabling the study of human mucosal microbiota during lengthy interventions involving multiple sampling timepoints [11,12,13]. One of the most broadly applied systems is the Mucosal Simulator of Human Gastrointestinal Microbial Ecosystem (M-SHIME^®^). Briefly, this model applies mucin agar-coated beads that mimic intestinal microcosms and are immersed in a bioreactor that was previously inoculated with human fecal microbiota [12]. A fraction of the microbial community from the fecal inoculum in the M-SHIME^®^ adheres to the microcosms and is considered representative of the human intestinal microbiota capable of colonizing the mucosal epithelial lining. In contrast, the luminal microbiota in the SHIME^®^ refers to the microbial ecosystem that establishes itself in the liquid phase of the bioreactors, either in the presence or absence of microcosms.

Validation studies have demonstrated that the M-SHIME^®^ system allows to generate and sustain an intestinal mucosal microbiota composed of taxa typically residing in human mucosa and distinct from the luminal microbiota [14,15,16].

Since its development and validation, the M-SHIME^®^ has been used in multiple studies exploring the responses of mucosal microbiota to a variety of nutrients, supplements, and interactions with ingested microorganisms, such as probiotics or pathogens [17]. Interactions with probiotics may be of particular interest as many of them, including *Lacticaseibacillus rhamnosus* GG (LGG) show not only mucous adherent properties, but have been found to modulate the composition of the human fecal microbiota [18,19]. Hence, such probiotics have the potential to also affect the mucosa-associated microbiota.

Other interventions affecting the composition of mucosal microbiota include changes in the diet. Multiple human studies have demonstrated that dietary components (both nutritive and non-nutritive) could affect the intestinal microbial community [20,21], although only a limited number focused particularly on mucosal microbiota composition [22,23,24,25,26]. A shared limitation of human studies in mucosal microbiota research is that sampling was limited to up to two biopsies per participant. Therefore, examining the effects of nutritional interventions in the M-SHIME^®^ could contribute to a broader understanding of how the human mucosal microbial community responds to dietary macronutrient intake and probiotic supplementation.

Since the mucosa-adherent microbiota in both humans and the M-SHIME^®^ develops through contact with the luminal content, documenting associations between the communities residing in each environment is valuable. Therefore, this study reported the response of the mucosal (M-SHIME^®^) microbiota to dietary macronutrient levels and LGG supplementation, correlated these responses with changes in the luminal microbiota (L-SHIME^®^), and discussed them in the context of human studies.

This study expands current knowledge of the behavior of human intestinal microbiota (both mucosal and luminal) during simulated dietary and probiotic interventions. It contributes to the understanding of correlations between luminal and mucosal microbiota, which have not been broadly discussed in the available literature.

To date, to our knowledge, no other published studies have investigated the effects of complex dietary interventions on simulated human microbiota in the M-SHIME^®^.

## 2. Materials and Methods

### 2.1. Design of the Study

A detailed description of this study is available from a previous publication [27], which reported a subset of data from the same experiment that led to the results presented here. Therefore, this section gives a short overview of the experimental design, aligned with the described dataset.

The SHIME^®^ (ProDigest, Gent, Belgium) setup consisted of ten bioreactors. Only two bioreactors were not inoculated with microbiota (joined stomach and ileum vessels). Another two bioreactors simulated the proximal colon, and the remaining six the distal colon. The content of each stomach/duodenum was pumped into a single proximal colon bioreactor and from there to three distal colon compartments, giving two separate arms. One arm was run in the L-SHIME^®^ mode, whereas the second was run in the M-SHIME^®^ mode. The M-SHIME^®^ was distinct from L-SHIME^®^ as it included 30 mucin beads in each distal colon bioreactor, and also a lower concentration of mucin was added to the feed media (2 instead of 3 g/dm^3^). The system was run under standard conditions (the pH in particular compartments of the simulated gastrointestinal tract, as well as time, and duration of the between-compartment flow of the digestive content) recommended by the manufacturer in the SHIME’s^®^ manual.

The system was inoculated with the feces of a 39-year-old healthy female volunteer, according to the SHIME^®^’s manufacturer’s instructions. Microbiota from a single donor was selected for system inoculation rather than a mixture of microbiota from multiple donors, as previous studies have shown that the establishment and stability of microbial communities in bioreactors may be affected by combining microbial strains that do not naturally cohabit [28]. This approach is consistent with established SHIME^®^ practices and manufacturer recommendations, which advise using microbiota from a single donor per experimental run/replicate. In the context of the current and similar studies, this implies that the observed responses may be specific to the applied donor microbiota.

After inoculation, the system was allowed to stabilize for 14 days using a standard protocol and standard feed media. Then a 28-day experiment was run.

Sampling from SHIME^®^ was performed 11 times throughout the experiment:

- Once prior to the start of interventions, immediately after the stabilization phase (SL—luminal sample and SM—mucosal sample);
- Five times during the combined probiotic and dietary intervention (on intervention days 3, 6, 8, 10, 12; samples L1–5 and M1–5 for luminal and mucosal SHIME^®^, respectively);
- Five times during the dietary intervention only (on intervention days 16, 18, 22, 25, 28; samples L6–10 and M6–10 for luminal and mucosal SHIME^®^, respectively).

**Figure 1:**
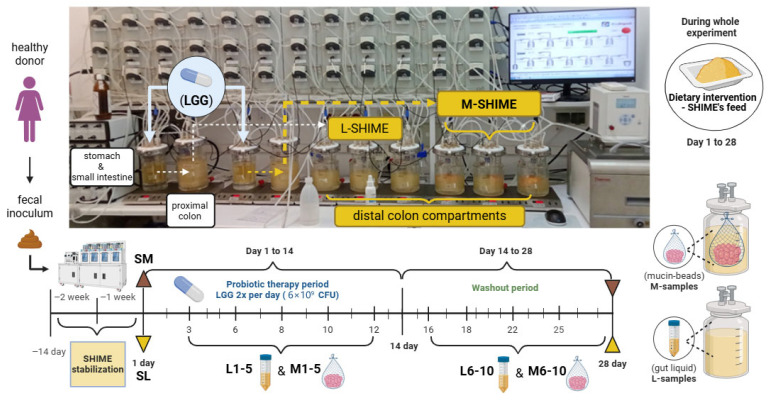
A schematic representation of the study and sampling scheme. The picture of SHIME^®^ was taken during the actual experimental run. The remaining elements were created with Biorender.com.

Samples were collected from each of the distal colon compartments—liquid from the L-SHIME^®^ arm and 15 mucosal beads from the M-SHIME^®^ arm. The mucosal beads were washed twice in sterile potassium phosphate buffer (pH 7.0) before DNA extraction, following the manufacturer’s protocol. The washing step is designed to limit the amount of luminal microbiota, shifting the balance towards the mucin-adherent microorganisms. The design of the study, together with the sampling scheme, is summarized in Figure 1. The experimental design was approved by the Research Ethics Committee at Jan Dlugosz University in Czestochowa.

### 2.2. Interventions

Applied interventions included a 28-day SHIME^®^ feed modification and 14-day supplementation with probiotics. Both interventions were administered in parallel from the beginning of the experimental phase.

The SHIME^®^ feed modification was designed to reflect the nutrient content of a balanced standard diet. It was based on the manipulation of the proportions of nutrients in a standard medium without introducing others, such as fat, which is not included in a standard feed. Daily nutrient inputs were adjusted according to the menus provided to the fecal donor who supplied the inoculum for the SHIME^®^ system. These menus were composed and supplied by a professional dietary catering company, and the nutrient contents were calculated as described in our previous work [27]. Detailed information on the menus, their nutrient content and SHIME^®^’s feed composition, as well as the methodology used for SHIME^®^ nutrient calculation, has been published previously [18,29]. In short, the concentrations of standard SHIME^®^ feed media components were manipulated to achieve a representation of food residues that could have reached the colon of the volunteer participating in the study. Animal protein, non-animal protein, sugars and soluble fiber dietary residue contents were simulated by adjusting special peptone, yeast extract, glucose, and a sum of xylan, gum arabic, pectin and starch concentrations in SHIME^®^ feed, respectively. The standard feed medium does not include fat-derived components, which was consistently followed in the current study.

For the probiotic intervention, LGG (ATCC 53103) was selected due to widespread knowledge on its microbiota-modulating properties and documented health benefits [30]. The LGG intervention was introduced into SHIME^®^ twice daily, manually into the stomach bioreactor, once its filling cycle was complete, ensuring that LGG underwent a full digestion process. The LGG supplement was a commercial powdered formulation, labeled as containing 6 × 10^9^ CFU per capsule and additives such as maltodextrin, hydroxypropyl methylcellulose and magnesium salts of fatty acids. The supplement was purchased in a local pharmacy. The contents of three capsules were suspended in a small volume of sterile water and divided evenly to inoculate both L- and M-SHIME^®^ arms.

### 2.3. Preparation and Analysis of Samples

Freshly collected samples were subjected to DNA extraction. Extraction was performed for each of the three liquid samples of the L-SHIME^®^ arm and the mucin-coated beads from the M-SHIME^®^ arm. The quality of DNA isolates was monitored by means of polymerase chain reaction (PCR) followed by gel electrophoresis and spectroscopy. Then, DNA isolates were pooled prior to 16S rRNA V3–V4 region Next Generation Sequencing (NGS), resulting in 11 samples per experimental arm. Detailed information on DNA extraction, NGS and metagenomic data processing has been described in our previous publication [27].

### 2.4. Statistical Analysis

The NGS data, as available from an open repository [29,31,32], were used to calculate α-diversity indices (Shannon, Simpson, and Chao1), the Firmicutes-to-Bacteroidetes ratio (F/B ratio), and the relative abundances at phylum, genus and operational taxonomic unit (OTU) level. These data were then used for the statistical analysis described below.

The following statistical procedures were applied:

1. Principal Coordinate Analysis (PCoA) based on the calculation of Jaccard distance was used to preliminarily assess the structure of M- and L-SHIME^®^ datasets and identify any outliers (OTU level);
2. The Wilcoxon signed-rank test was applied to assess differences in general microbiota structure between L- and M-SHIME^®^ (α-diversity indices and F/B ratio);
3. Spearman’s correlation analysis was performed to:
  - Assess whether fluctuations in genera abundance in L-SHIME^®^ triggered corresponding changes in M-SHIME^®^;
  - Conduct sensitivity analysis to identify potential lagged responses of M-SHIME^®^ microbiota to fluctuations in dietary nutrient intake, following previously described methodology [18];
  - Assess associations between dietary macronutrient intake and microbiota structure (at α-diversity, F/B ratio and genus level) in M-SHIME^®^ after adjusting for a parallel LGG intervention.
4. Linear model (LM) followed by type III Analysis of Variance (ANOVA) constituted the main analysis. This analysis was performed using data encompassing the whole microbiota at the phylum level. It aimed to evaluate differences in the microbiota responses to interventions between L- and M-SHIME^®^. The first two principal components (PC) of microbiota abundance and dietary macronutrient intake (animal and non-animal protein, sugars, soluble fiber) data, derived from separate principal component analyses (PCAs), were included in the model described by dependence (1):

PC1_micro or PC2_micro ~ Environment × (Probiotic + PC1_diet + PC2_diet) (1)

where

PC1_micro and PC2_micro—PCs of phylum-level microbiota abundances,

PC1_diet and PC2_diet—PCs of the macronutrient intake,

Probiotic—presence/absence of LGG supplementation,

Environment—SHIME^®^ compartment (L-SHIME^®^ vs. M-SHIME^®^).

Type III ANOVA was used to test the main and interaction effects of variables included in the model.

Analyses numbered 1, 3 a and c, and 4 were performed in R (RStudio; version 2025.09.0+387; Posit, Boston, MA, USA). The R scripts were generated with assistance from Generative Artificial Intelligence (ChatGPT ver. 5.1; OpenAI, San Francisco, CA, USA). User-verified versions of the scripts were deposited in an open repository [31]. The remaining statistics were performed in Statistica ver. 13 (StatSoft, Kraków, Poland). The compositional data were center log ratio-transformed (CLR) [33] before all analyses, with the exception of PCoA. The outlier identification based on PCoA analysis relied on the visual inspection of the plot. The outliers were termed as samples that were clearly separated from the main sample clusters in the ordination space. Given the inherent complexity and variability of human microbiome data, exploratory visualization approaches such as PCoA applied here are commonly used to identify atypical community profiles. All statistical tests were performed assuming a significance threshold of 0.05. To correct for multiple comparisons, either the Benjamini–Hochberg false discovery rate (FDR) or Bonferroni correction was applied (see Section 3). The interpretation of the results was primarily based on corrected *p*-values. For correlation analyses, in addition to statistical significance, the strength of associations was also considered, with Spearman’s correlation coefficients of absolute value greater than 0.7 interpreted as strong associations. Adjustment for the probiotic intervention in analysis 3 c was performed using residuals derived from linear models including probiotic intervention as a binary covariate prior to correlation analysis.

## 3. Results

### 3.1. The Composition of Mucosal Microbiota in M-SHIME^®^ During the Experiment

**Figure 2:**
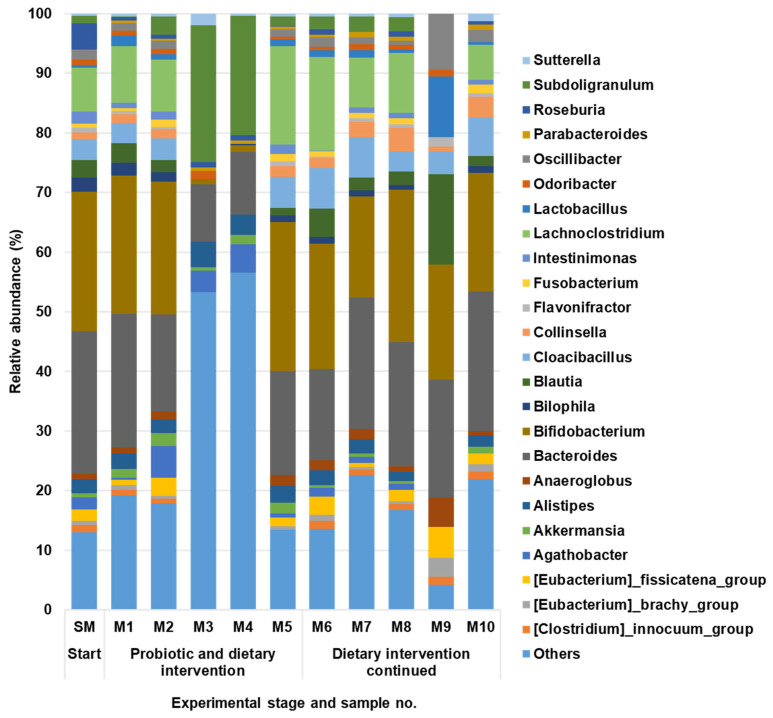
Relative abundance of the most prevalent bacterial genera (MPG, 24 genera detected in 8 out of 11 samples) in M-SHIME^®^ during the experiment.

The genus-level composition of M-SHIME^®^ microbiota is shown in Figure 2. During the experiment, fluctuations of the microbial community were noted, but without any trends. Overall, the majority of samples (8 out of 11) contained 24 genera at the detectable level (the most prevalent genera, MPG), with *Lachnoclostridium*, *Bifidobacterium* and *Bacteroides* dominating and each accounting for up to 16–25% and jointly constituting between 47 and 59% of the total bacterial population.

Three samples deviated from this general pattern: M3 and M4 taken during the parallel probiotic and dietary intervention, and M9 during the continued dietary intervention alone. Samples M3 and M4 were characterized by a low relative abundance of *Lachnoclostridium* and *Bifidobacterium* and a high proportion of *Subdoligranulum*, whereas M9 exhibited low proportions of *Lachnoclostridium* and higher proportions of the former genus *Lactobacillus* and *Blautia* compared with all the other samples.

### 3.2. Comparison of the Mucosal and Luminal Microbiota Composition in SHIME^®^

**Figure 3:**
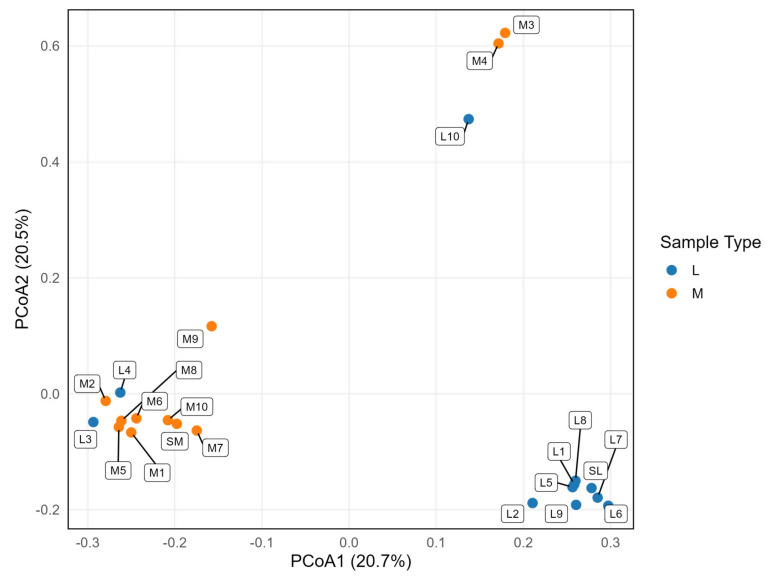
Principal coordinate analysis (PCoA) based on Jaccard distance for M-SHIME^®^ (M) and L-SHIME^®^ (L samples) at the operational taxonomic unit level.

The PCoA analysis based on the Jaccard distance (Figure 3) revealed that both L and M samples created distinct clusters at the OTU level. Nevertheless, upon visual inspection, several outliers were identified. For the M-SHIME^®^, the outlying samples were M3, M4 and M9, whereas for the L-SHIME^®^ they were L3, L4 and L10. Further examination of the relative taxonomic abundances in the outlying samples confirmed that they featured distinct microbiota profiles compared with other samples in the respective clusters, supporting the PCoA outcomes. Therefore, the outlying samples were excluded from further analyses.

**Figure 4:**
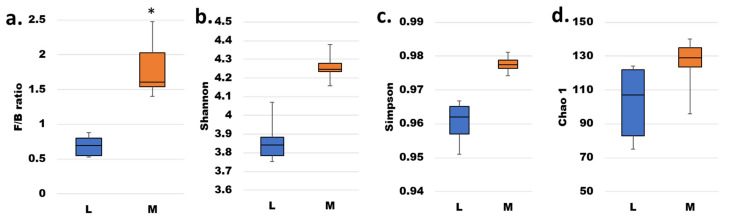
Firmicutes-to-Bacteroidetes (F/B) ratio (**a**) and Shannon, Simpson and Chao1 α-diversity indices (**b**–**d**, respectively) of L-SHIME^®^ (L) and M-SHIME^®^ (M) samples. Paired L and M data (n = 7) were analyzed by means of Wilcoxon’s signed-rank test at a significance level of 0.05, with Bonferroni correction applied to the α-diversity indices. Statistically significant differences were marked with an asterisk (*).

Differences in microbial populations between the mucosal and luminal microbiota samples were apparent already at the phylum level. The abundance of the two most dominant phyla, Firmicutes and Bacteroidetes, ranged from 38 to 47 and 19 to 27% in M-SHIME^®^ samples and from 28 to 42 and 43 to 54 in L-SHIME^®^ samples, respectively (data for all samples available from [31]). Consistent with the PCoA findings, the F/B ratio and α-diversity indices differed between the samples, with both measures being higher in M- than in the L-SHIME^®^ (Figure 4). Neither of the triple α-diversity comparisons survived the Bonferroni correction, despite displaying significance before the correction. On the other hand, the difference in F/B ratio between the M- and L-SHIME^®^ was statistically significant.

**Figure 5:**
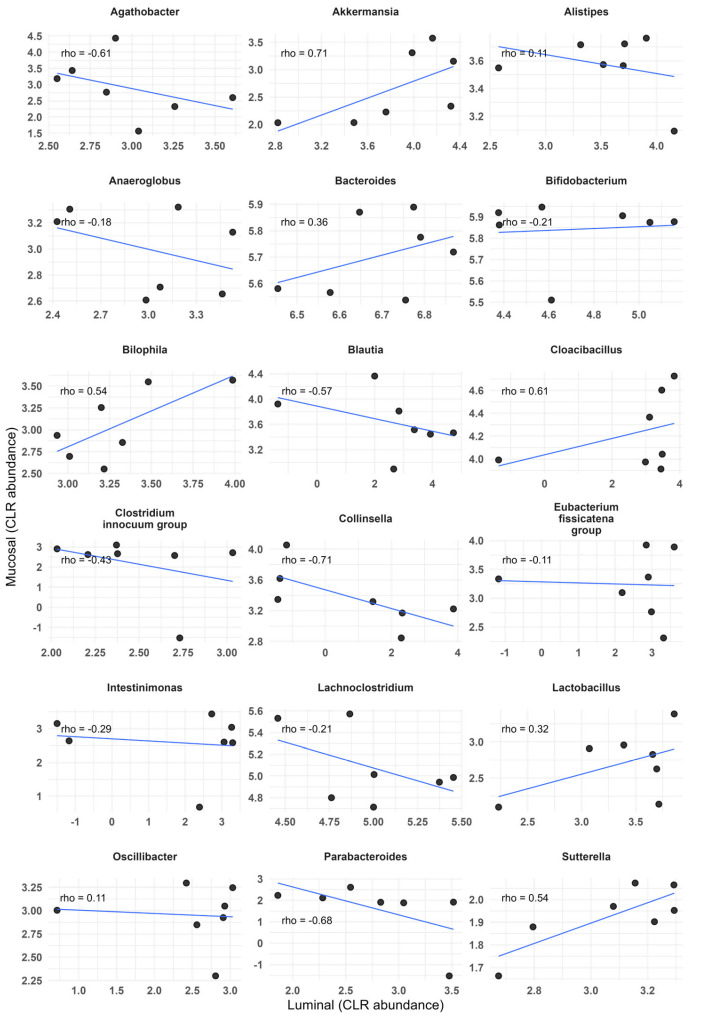
Spearman’s correlation between the center log ratio-transformed abundance of the 18 most prevalent genera (MPG) in the M- and L-SHIME^®^ samples. No significant correlations were found.

At the genus level, 14 taxa that were detected in M did not appear in L samples. All of these genera were either detected in the sample of fecal inoculum (four of fourteen) or represented well-known members of the typical human microbiota. On the other hand, only five genera detected in L were not found in M samples. Despite these differences, 18 out of the previously identified 24 MPG were shared between the L- and M-SHIME^®^. Interestingly, the CLR-transformed relative abundances of the mentioned 18 MPG showed low correlation between L- and M-SHIME^®^ environments (Figure 5), with no statistically significant associations found. High correlation coefficients (>|0.7|) were identified only for *Akkermansia* (positive) and *Collinsella* (negative, Figure 5).

### 3.3. The Impact of Applied Probiotic and Dietary Interventions on the Mucosal Microbiota in SHIME^®^

The strongest response of the microbiota was observed when the macronutrient intake from 24 h or as an average of 4 days before the sampling was included (sensitivity analysis, data available in an open repository [31]). Because the mucosal beads were sampled and replaced after 4–5 days, a 4-day mean nutrient intake was considered in further analyses. For the L-SHIME^®^ compartment, a 12-day mean nutrient intake was applied as previously established [18].

**Figure 6:**
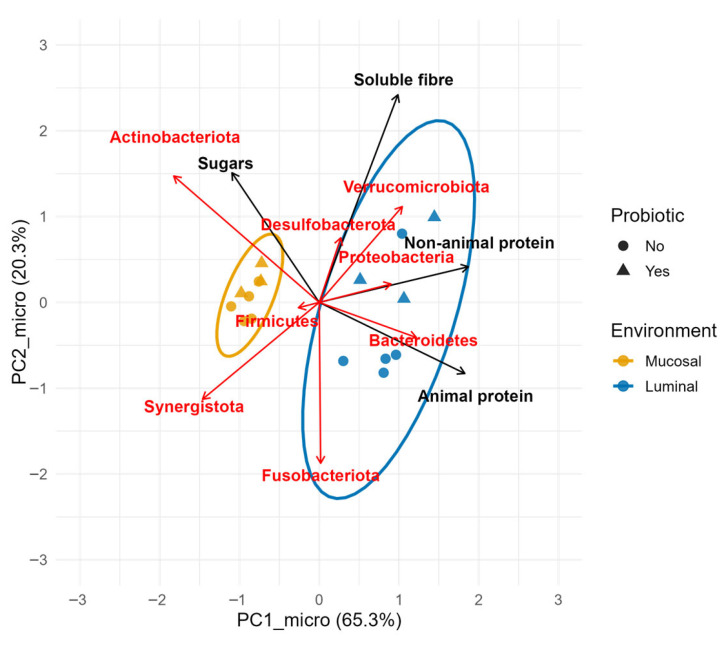
Biplot of principal components of bacterial phyla in L (Luminal) and M (Mucosal) SHIME^®^ environments. Points represent samples, red vectors show rescaled phylum loadings, and black vectors express overlaid loadings of dietary macronutrients projected as supplementary variables into the microbiota PCA space. The direction of each vector reflects its correlation with the PCA axes, whereas vector length indicates the strength of this association. Ellipses mark 95% confidence intervals around samples.

The impact of the applied interventions on the full microbiota structure at the phylum level, as revealed by the linear modeling of the PCA scores for microbiota and macronutrients (separate PCA, overlaid vectors), was visualized in Figure 6, and the full output of the analysis was contained in the open repository [31]. According to type III ANOVA, the environment had a significant effect on PC1_micro (explaining ~61% of the variance). Verrucomicrobiota, Proteobacteria and Bacteroidetes dominated the L-SHIME^®^, whereas Actinobacteriota, Firmicutes and Synergistota dominated the M-SHIME^®^ environment. On the other hand, no similar effect was found along PC2_micro. The ellipses marking 95% confidence intervals for M- and L-SHIME^®^ samples showed leveled centroids along PC2_micro, indicating no environmental separation on this axis. However, samples from the L-SHIME^®^ were characterized by a markedly greater dispersion compared with the M-SHIME^®^. Despite the difference in dispersion, both ellipses exhibited nearly parallel major axes, suggesting that the underlying structure of inter-individual variability was similar across mucosal and luminal environments.

Both interventions seemed to influence mainly PC2_micro (~10% and 14% variance explained by PC1_diet and Probiotic, respectively). In addition, a very strong interaction effect was noted between PC1_diet and Environment for PC2_micro (nearly 52% of the variance explained), which was predominantly attributed to changes in the concentrations of both animal and non-animal protein. Notably, non-animal protein correlated positively with the relative abundance of Proteobacteria, whereas animal protein promoted Bacteroidetes.

Soluble fiber and sugars loaded predominantly onto the PC2_diet, which accounted for only ~24% of the variability in nutrient intake (vs. 56% of PC1_diet), seemed to have a smaller effect on microbiota fluctuations (the output of the analysis is available in open repository [31]). However, among all microbial phyla considered, sugars most strongly promoted Actinobacteriota, which dominated the M-SHIME^®^ community.

**Figure 7:**
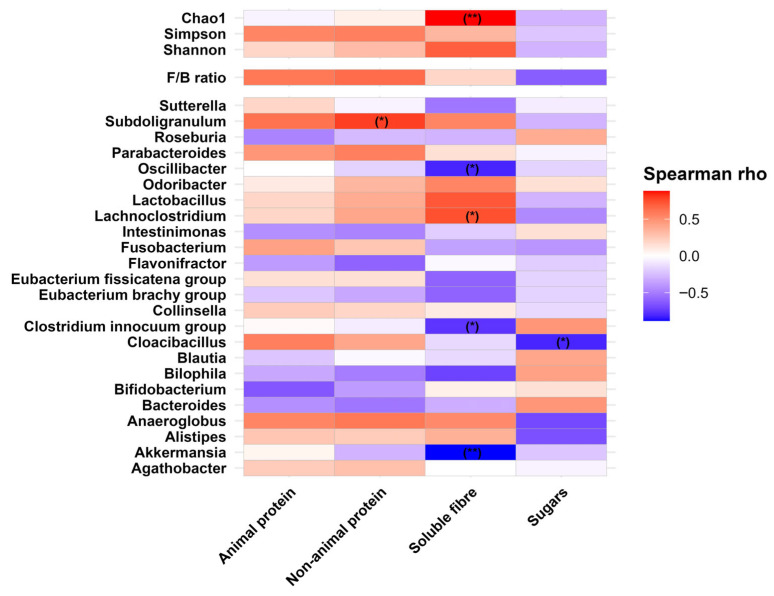
Heatmap of Spearman’s correlation coefficients between dietary nutrient intakes and probiotic intervention-adjusted residuals for 1. the α-diversity indices, 2. the Firmicutes-to-Bacteroidetes (F/B) ratio, and 3. the center log ratio-transformed relative abundance of the most prevalent bacterial genera (MPG) in M-SHIME^®^ samples. Statistical significance was marked with ** *p* < 0.01, and * *p* < 0.05 for raw *p*-values in brackets. Neither of the *p*-values remained significant after Benjamini–Hochberg false discovery rate (FDR) correction. FDR corrections were applied for each of 1. α-diversity indices, 2. F/B ratio, and 3. MPG correlations, separately.

To understand how individual macronutrients influenced microbiota structure at α-diversity, F/B ratio and MPG levels in the M-SHIME^®^, Spearman’s correlation analysis on probiotic intervention-adjusted residuals was performed (Figure 7). Several *p*-values indicated nominal significance (<0.05), including positive correlations between non-animal protein and *Subdoligranulum*, soluble fiber and *Lachnoclostridium*, as well as soluble fiber and Chao1 index. However, none of these correlations remained significant after FDR correction. Despite this, all three were characterized by a Spearman’s *ρ* > 0.7. Similarly, there were no negative correlations which survived FDR correction, although few were comparatively strong (Spearman’s ρ < −0.7, correlations between soluble fiber and three genera: *Oscillibacter*, *Clostridium innocuum* group and *Akkermansia*, as well as between sugars and *Cloacibacillus*).

## 4. Discussion

The diversity of mucosal-associated bacteria in humans is typically comparable to that found in the intestinal lumen, but these communities differ in composition [4]. Importantly, functional differences can exist within the same bacterial species, which are reflected in transcriptional and metabolomic profiles. Therefore, the term “mucosal-associated” is a spatiotemporal description that also encompasses distinct functional and metabolic activities and interactions with the immune system, enabling microorganisms to survive in this niche. The relative proximity of these mucosal-associated organisms to the epithelial border increases the likelihood of interactions with the intestinal immune system, from epithelial cells to immune cells in the underlying lamina propria [34].

This is important for elucidating the effects of dietary components or probiotic therapy on microbiota modulation and, consequently, on human health. In vitro studies offer the opportunity to distinguish these major fractions of the gut microbiota. The M-SHIME^®^ is a validated platform for studying the dynamics of human mucosal microbiota. In particular, studies have shown that compared with the L-SHIME^®^, M-SHIME^®^ promotes the enrichment of Firmicutes at the expense of Bacteroidetes and Proteobacteria [15,16,35]. This pattern is in line with the observations reported in the present study (Figure 6).

However, contemporary human studies frequently note that although the Firmicutes are a dominant phylum in the human colonic biopsies, their relative abundance is typically lower than in fecal samples [2,3]. On the other hand, mucosal biopsies tend to be enriched in Proteobacteria and Bacteroidetes, partly driven by the expansion of the genus *Bacteroides* [2,3,36,37,38,39,40]. In the current study, *Bacteroides* was indeed one of the three major genera present in the M-SHIME^®^ samples (Figure 2). However, its relative abundance remained much lower compared to the L-SHIME^®^ (15–24% vs. 40–52%), as presented in our previous report from the experiment that also led to the current work [18,29].

On the other hand, we have noted that the phylum Actinobacteriota was strongly associated with the M-SHIME^®^ (Figure 6), primarily due to the high abundance of *Bifidobacterium* (Figure 2). This aligns with reports from human studies, suggesting that Actinobacteriota may preferentially populate the mucosal layer of the intestine, potentially due to an increased oxygen availability compared to lumen [36,37,38]. However, a contrasting outcome was reported in another in vitro study in which the M-SHIME^®^ community exhibited a significantly lower abundance of *Bifidobacterium* than the corresponding L-SHIME^®^ compartment [35].

Relatively little information is available on correlations between luminal and mucosal microbiota. One exemplary study where the authors correlated colonic biopsies and fecal samples from human donors reported high (correlation coefficient > 0.7), positive and statistically significant relationships for five genera, including *Akkermansia* and *Collinsella* [2]. In contrast, we did not observe any statistically significant correlations for the abundance of MPG between the L- and M-SHIME^®^. Still, the correlation coefficients for *Akkermansia* and *Collinsella* indicated strong positive and negative relationships, respectively (Figure 5). Differences in the study design may partly explain this discrepancy. While Ringel et al. correlated cross-sectional pairs of mucosal and luminal samples collected from different individuals [2], in our work, all samples originated from the same experimental system, sampled repeatedly over time.

Any discrepancies between human and in vitro studies examining dependencies between luminal and mucosal microbiota structure may be attributed to limitations of current in vitro models. Even state-of-the-art systems lack the complexity of the human organism, including its absorption and secretory activity.

The mucosal-associated microbiota is thought to be less affected by the intestinal contents compared to the luminal community [38]. In line with this, we observed markedly lower dispersion of M-SHIME^®^ compared to the L-SHIME^®^ samples (Figure 6), which suggested a more constrained and less variable response of mucosal microbiota to applied interventions.

On the other hand, multiple human studies have shown that the mucosal-associated microbiota does respond to a variety of dietary changes and supplementation. For example, a 12-day supplementation with a probiotic beverage resulted in the colonization of colonic mucus by LGG, which was detectable in some biopsies for up to 14 days after beverage discontinuation, despite the strain being absent from the fecal samples collected two days prior to the biopsies [41]. Apart from this evidence of LGG persistence in the gastrointestinal tract, little is known about its influence on the mucosal intestinal microbiota of humans. A recent pilot study, in which biopsies from 13 ulcerative colitis patients undergoing 2-week supplementation with LGG were sampled, showed a reduction in α-diversity due to a decrease in the abundance of such genera as *Clostridium*, *Phocea*, *Intestinibacter*, *Lacrimispora*, and *Faecalicatena*, and several particular species, including *Coprococcus catus*, *Phocea massiliensis*, *Bacteroides thetaiotaomicron*, *Bacteroides uniformis*, and *Intestinibacter bartlettii*, all belonging either to Firmicutes or Bacteroidetes [42].

In our study, we also observed that LGG supplementation induced a shift in the microbiota, both mucosal and luminal (Figure 6); however, the main trends that were observed showed an increase in Desulfobacterota and Verrucomicrobiota. The probiotic supplementation did not seem to affect the α-diversity, whereas the relative abundance of Firmicutes and Bacteroidetes did not display any consistent pattern [31].

More data are available on the effect of dietary interventions on the mucosal microbiota. For example, a high-protein, isocaloric diet administered for 3 weeks did not alter the overall composition of mucosal microbiota in obese adults; however, the authors noted a shift in the bacterial metabolism towards increased protein degradation [43]. In another work, authors evaluated the association of a Healthy Eating Index (HEI)-2005 score [44] with the composition of mucosal colonic microbiota (97 specimens) collected from 34 Americans aged 50–75 years who were at risk of developing a colorectal tumor and who underwent a clinically ordered colonoscopy [22]. This study has found numerous associations, such as a positive correlation of HEI with the abundance of *Subdoligranulum* and *Parabacteroides*, high fruit intake encouraged *Faecalibacterium*, *Akkermansia*, *Parabacteroides* and *Roseburia*, greater total grain and sugar consumption favored *Escherichia*, while milk and soy beverage consumption was positively correlated with the abundance of *Faecalibacterium* and *Fusobacterium*. In addition, vegetable and high saturated fat and calorie intake from solid fats, alcoholic beverages and added sugars promoted *Fusobacterium*. On the other hand, HEI score, fruit, milk and soy beverage intake, as well as the calorific value of dietary solid fats, alcohol, and added sugar were found to be positively associated with the α-diversity (Shannon index). Although significant negative correlations were also reported, their interpretation is more complex. Dietary macronutrients do not directly inhibit particular bacterial groups; rather, they promote other members of the complex microbial community, which then can suppress the growth of others. For this reason, in the present discussion, we focused on positive associations between macronutrients and microbiota.

A continuation of the previously mentioned study [22] using a matching design examined the influence of dairy consumption on the mucosal microbiota [24]. The abundance of *Faecalibacterium* and *Akkermansia* was positively correlated with milk intake. In addition, *Faecalibacterium* seemed to be encouraged by dairy. Similarly, α-diversity was positively associated with the consumption of milk and dairy products but decreased with cheese intake. An increase in cheese consumption is accompanied by a higher intake of animal fat. Although fat is often a neglected macronutrient in microbiota research, it is not present in the feeding media for the SHIME^®^, and was shown to matter to microbiota much less than the intake of fiber [45]; some works have demonstrated that reducing its consumption could exert a beneficial effect on microbiota. For example, an additional analysis of the same American dataset used in previously mentioned studies [22,24], revealed that the profile of fatty acids in the consumed diet caused significant changes in the mucosal microbiota. The abundance of *Sutterella* increased with the intake of total fatty acids as well as mono- and poly-unsaturated fatty acids, whereas *Tyzzerella* and *Fusobacterium* seemed to be encouraged by saturated fat and only *Tyzzerella* was associated with the intake of trans-fatty acids [26]. In another study involving 36 normal and overweight Chinese adults aged 45–65 years with a high habitual fat intake (>40% energy from fat), nine participants remained on a high-fat diet (control), and nine were given a low-fat dietary intervention (treated) for four months. The dietary reduction in fat exerted some beneficial effects on mucosal microbiota. Specifically, the α-diversity (Chao1 index) increased both within and between groups. The authors also reported that Firmicutes and Bacteroidetes had clearly higher abundances in the treated group compared to the control at the expense of Proteobacteria and Actinobacteriota. However, these results should be interpreted with caution. The authors did not provide data on the microbiota composition in each group before intervention (except for α-diversity), and full information on the calorific value and proximate composition was missing from the manuscript, making it difficult to determine whether the observed changes were attributable specifically to fat reduction or to other dietary factors.

Some works evaluated the effect of dietary compounds other than basic macronutrients on the mucosal intestinal microbiota of humans, such as carotenoids and human milk oligosaccharides (HMO). For example, in a study where American participants received dietary counseling to follow either a Mediterranean or high-HEI diet for 6 months, the change in dietary pattern itself did not affect the structure of the mucosal colonic microbiota. However, participants with greater intake of carotenoids were characterized by an increase in the relative abundance of *Prevotella*. Interestingly, carotenoid intake was not associated with carotenoid levels in excreted feces, leading the authors to the conclusion that the mucosal microbiota may play an important role in the bioavailability of these nutrients [25]. On the other hand, a study where 58 patients with Irritable Bowel Syndrome were supplemented for 4 weeks with HMO: 2′-O-fucosyllactose and lacto-N-neotetraose or a placebo reported that the treatment did not affect the β-diversity (gut microbiota composition between samples) of the mucosal microbiota [40]. However, it did increase a relative abundance of *Bifidobacterium* ssp., especially *Bifidobacterium adolescentis* and *Bifidobacterium longum*.

Human studies rarely investigate the effects of single macronutrients; rather, they focus on dietary quality or specific food groups, making it difficult to disentangle the impact of individual nutrients. However, these studies consistently demonstrate that macronutrients may play an important role in shaping the human mucosal microbiota, and this is confirmed by the current study. Although our results were underpowered due to the application of a conservative FDR correction, a few strong correlations between macronutrient intake and MPG abundance were observed. These results arguably supported findings from human studies. For example, the abundance of *Subdoligranulum* and *Parabacteroides* was positively correlated with animal and non-animal protein, as well as soluble fiber content (Figure 7), aligning with previously reported associations of these genera with HEI scores [22]. Moreover, *Fusobacterium* increased with animal and non-animal protein, which is consistent with a previous observation from a human study that associated this genus with milk and soy beverage intake [22]. *Sutterella*, which was promoted by animal protein in our study, was also encouraged by total fatty acid intake—a dietary component typically positively correlated with animal protein consumption [22,24]. In addition, the abundance of *Roseburia* and *Bifidobacterium*, which were found to be enriched in the mucosa of humans consuming high volumes of fruits and supplementing HMO, respectively [22,40], were found to correlate weakly but positively with sugar in M-SHIME^®^ feed media. Although modest, these associations may suggest that certain nutrient–microbiota relationships observed in vivo can be at least partially reproduced in the SHIME^®^ model.

To date, to our knowledge, no other published studies have investigated the effects of complex dietary interventions on simulated human microbiota in the M-SHIME^®^. Available reports have instead focused on specific substances, such as lactose [46,47], HMO [46,47,48], prebiotic or potentially prebiotic fibers (e.g., fructooligosaccharides—FOS from green and gold kiwifruit [49], soluble corn fiber [50], inulin [51,52], fibers rich in naturally occurring bound polyphenols [51], psyllium [51], galactooligosaccharides—GOS [48] and carrot-derived pectin extract enriched for rhamnogalacturonan I [53]), ω-3 polyunsaturated fatty acids [54], linoleic acid [35], plant extracts or oils (e.g., extract from *Vitis vinifera* [55], proanthocyanidin-rich aronia extract [56], cranberry extract [57], *Buglossoides arvensis* oil rich in stearidonic acid [58]) or particular supplements such as D,l-lactide-co-glycolide particles [59] or formulations containing, among other ingredients, citrus phenols and amino acids (l-proline, l-serine, l-cysteine, l-threonine) [60].

Only a few studies examined the fate of food items introduced into the M-SHIME^®^. Examples include freeze-dried cranberry powder introduced into the system directly with the feed [61], pre-digested yeast and sourdough bread [62] or pre-digested oat ingredients [63].

All of the interventions applied in the mentioned M-SHIME^®^ studies were shown to exert a significant impact on the microbiota structure. Although the featured nutrients, foods, and supplements differ from macronutrients studied here, their dietary intake could be related, enabling a discussion.

Several taxa that responded with an increase in relative abundance to interventions in the M-SHIME^®^ in previous studies were identified among the MPG within the current work. For example, *Subdoligranulum* was previously reported to be encouraged by linoleic acid [35], which is commonly present in plants, including those rich in protein, such as soy or nuts. In the current study, its abundance correlated particularly strongly with the content of non-animal protein in M-SHIME^®^ feed. In other studies, the former genus *Lactobacillus* seemed to be stimulated by FOS, oat ingredients, and cranberry extract, similarly to *Bifidobacterium*, which, in addition, correlated positively with soluble corn fiber and pectin extract from carrots enriched for rhamnogalacturonan I [49,50,53,57,63]. Here, the former genus *Lactobacillus* responded weakly but positively to soluble fiber, whereas *Bifidobacterium* showed a similarly weak positive association with sugars—nutrients linked to dietary intake of the foods and fibers mentioned in the cited literature.

Other taxa related to the MPG included members of the Lachnospiraceae family and the genus *Akkermansia*. Lachnospiraceae increased in abundance in response to fibers rich in polyphenols, inulin and freeze-dried cranberries, whereas *Akkermansia* was promoted by ω-3 polyunsaturated fatty acids and *Buglossoides arvensis* oil, rich in stearidonic acid [51,54,58,61]. In the present study, the abundance of *Lachnoclostridium*—a genus belonging to the Lachnospiraceae family—correlated strongly with soluble fiber, while none of the nutrients in fat-deprived M-SHIME^®^ feed seemed to encourage *Akkermansia*.

Overall, the responses of the MPG observed in this work are consistent with findings from previous human and in vitro studies, despite differences in the type and complexity of dietary interventions applied.

## 5. Conclusions

This study demonstrated that the mucosal microbiota in the SHIME^®^ gave distinct responses to dietary intervention compared to luminal microbiota, including a smaller overall shift in microbiota composition. On the other hand, probiotic (LGG) supplementation induced comparable shifts in the microbial communities of both SHIME^®^ compartments. These observations support the necessity of extending the focus of the intestinal microbiota studies from luminal to also mucosal, especially since the mucosal microbiota may be a better predictor of pathological conditions, taking part in many important processes, including the alteration of nutrient bioavailability, immunomodulation and protection from pathogens.

Observed associations between dietary macronutrient intake and changes in the M-SHIME^®^ microbiota composition were largely consistent with patterns known from previous human studies, as well as in vitro research. Although the number of statistically robust associations was limited, the directionality of several key correlations (e.g., involving *Subdoligranulum*, *Parabacteroides* or *Fusobacterium*) aligned with in vivo studies. These results suggest that the M-SHIME^®^ allows at least partial reflection of the nutrient–microbiota relationships relevant to human mucosa.

Together, these observations confirm the value of the M-SHIME^®^ in studying the responses of human mucosal microbiota to dietary and supplement interventions. Given that this study is based on a single exploratory experiment, the findings should be interpreted as hypothesis-generating and require validation in independent studies involving microbiota from multiple donors. More research is also required to understand what changes in the mucosal microbiota are beneficial for human health, since most of the relationships between microbiota and pathological conditions were established based on studies of fecal communities. This part of the human microbiota seems to play a pivotal role in intestinal barrier integrity and immune function, including pathogenesis of conditions such as inflammatory bowel disease; therefore, studies with multiple sampling during prolonged interventions are essential to understand what period of time is required to induce beneficial changes.

## Data Availability

The full dataset that this report was based on is available under: https://zenodo.org/records/15348077 (last accessed on 20 December 2025) and https://zenodo.org/records/16751628 (last accessed on 20 December 2025; both datasets also shared with previous publications) and under https://zenodo.org/records/17910699 (last accessed on 16 January 2026; exclusively for the current report).

## Abbreviations

The following abbreviations are used in this manuscript:

ANOVA	Analysis of Variance
CFU	Colony-forming units
DNA	Deoxyribonucleic acid
FDR	False discovery rate
FOS	Fructooligosaccharides
F/B ratio	Firmicutes-to-Bacteroidetes ratio
GOS	Galactooligosaccharides
HEI	Healthy Eating Index
HMO	Human Milk Oligosaccharides
LGG	*Lacticaseibacillus rhamnosus* Strain GG
LM	Linear Model
L-SHIME^®^	Luminal Simulator of Human Gastrointestinal Microbial Ecosystem
MPG	Most prevalent genera
M-SHIME^®^	Mucosal Simulator of Human Gastrointestinal Microbial Ecosystem
NGS	Next Generation Sequencing
OTU	Operational taxonomic unit
PC	Principal Component
PCA	Principal Component Analysis
PCoA	Principal Coordinate Analysis
PCR	Polymerase chain reaction
rRNA	Ribosomal ribonucleic acid
SHIME^®^	Simulator of the Human Intestinal Microbial Ecosystem
